# Imaging features of extranodal involvement in paediatric Hodgkin lymphoma

**DOI:** 10.1007/s00247-018-4280-z

**Published:** 2018-12-05

**Authors:** Suzanne Spijkers, Annemieke S. Littooij, Paul D. Humphries, Marnix G. E. H. Lam, Rutger A. J. Nievelstein

**Affiliations:** 1 Department of Radiology and Nuclear Medicine, University Medical Center Utrecht/Wilhelmina Children’s Hospital, Heidelberglaan 100, 3584 CX Utrecht, The Netherlands; 2 Department of Specialist Radiology, University College London Hospital, London, UK; 3 Department of Radiology, Great Ormond Street Hospital for Children, London, UK

**Keywords:** 18F-fluoro-2-deoxy-D-glucose, Adolescents, Children, Diffusion-weighted magnetic resonance imaging, Extranodal lymphoma, Hodgkin lymphoma, Positron emission tomography/computed tomography, Whole-body magnetic resonance imaging

## Abstract

Detecting extranodal disease in paediatric Hodgkin lymphoma is of great importance for both treatment and prognosis. Different imaging techniques can be used to identify these extranodal sites. This pictorial essay provides an overview of imaging features of extranodal disease manifestation in paediatric Hodgkin lymphoma.

## Introduction

Childhood Hodgkin lymphoma is one of the most curable paediatric cancers, with long-term survival rates above 90% for early stage disease [1]. After diagnosing paediatric Hodgkin lymphoma, imaging has a central role in determining the extent of disease as well as in monitoring treatement and follow-up [2]. Detecting noncontiguous extranodal disease, implicating stage IV disease, is of great importance since this has implications for both the strategy of treatment and prognosis [1–3].

In current practice, 18F-fluoro-2-deoxy-D-glucose (FDG) positron emission tomography (PET) combined with computed tomography (CT) is the reference standard for both staging and follow-up of Hodgkin lymphoma [2]. Although FDG-PET/CT is the current preferred imaging modality, ultrasonography (US) and magnetic resonance imaging (MRI) are increasingly used in both staging and follow-up since a substantial dose of radiation is used in FDG-PET/CT [4]. Depending on the child’s age and weight, and the local imaging protocols, estimated doses have been described to be around 9.3±2.3 mSv [5]. Especially when taking into account that during treatment repeated imaging is required, children with Hodgkin lymphoma might be at increased risk of radiation-induced malignancies later in life [3, 6]. In recent literature, both whole-body MRI and FDG-PET/MRI have been investigated as alternatives to FDG-PET/CT for staging (adult) Hodgkin lymphoma, in order to reduce radiation dose to the patient [7–11].

Although several previous studies have addressed imaging techniques of different sites of extranodal involvement in adult Hodgkin lymphoma [12–14], to the best of our knowledge, no previous review has focused on providing an overview of the imaging features in children. Therefore, the purpose of this pictorial essay is to illustrate the imaging findings of extranodal disease involvement in paediatric Hodgkin lymphoma. Case material used in this essay was collected from the University Medical Center Utrecht, Erasmus University Medical Center Rotterdam and University College London Hospital. This use of case material was approved by the local institutional research ethics board.

## Extranodal disease

Extranodal disease manifestation is defined as noncontiguous infiltration of malignant lymphomatous cells in extralymphatic organs and is classified as stage IV disease [2]. Contiguous organ involvement is an extension of disease originating directly from a known affected nodal site (E-stage disease; indicating stage I, II or III disease). E-stage disease must be distinguished from stage IV disease during the staging process since E-stage disease requires a less extensive treatment schedule [1–3]. Hodgkin lymphoma can spread to almost every organ system, although some organs are more often affected than others. In Hodgkin lymphoma, the most common sites of extranodal infiltration are bone marrow, lung and liver [15]. In children, reportedly 15% of patients are diagnosed with extranodal disease at presentation [16]. Of note, splenic involvement is considered nodal disease as well as involvement of Waldeyer’s ring and thymus [2]. An overview will be given of imaging features of the most frequently occurring manifestations of extranodal disease in paediatric Hodgkin lymphoma. Additionally, Table 1 summarizes the main values and limitations for each imaging modality used in staging Hodgkin lymphoma whereas Table 2 shows criteria for detecting extranodal involvement.

**Table 1:** Advantages and limitations of different imaging modalities used to identify extranodal manifestations of Hodgkin lymphoma and overall sensitivity/specificity for staging

Modality	Advantages	Technical limitations	Diagnostic limitations	Reported sensitivity/specificity for staging Hodgkin lymphoma
Whole-body MRI^a^	- No ionizing radiation exposure - High spatial resolution - Excellent soft-tissue contrast - Advanced techniques (e.g., diffusion-weighted imaging) for better tissue characterization - Whole-body imaging	- Long examination time (young children often need sedation/anaesthesia) - No standard imaging protocols (e.g., variation in the included body areas or sequences used) - Motion artefacts (respirational/cardiac) - No criteria available for use in response assessment	- Involvement of nodal disease still based on size criteria - Difficult to distinguish malignant and benign disease on diffusion-weighted imaging (e.g., lymph nodes)	91%/99%
CT	- Widely available - Fast - Whole-body imaging	- Exposure to ionizing radiation - Inability to differentiate between active disease and residual mass - Use of intravenous contrast agents	- Involvement of nodal disease based on size criteria	87.5%/85.6%
FDG-PET	- High diagnostic accuracy - Detection of metabolic activity - Whole-body imaging	- Exposure to ionizing radiation - Low spatial resolution, unable to detect small lesions - Extensive patient preparation time - Long examination time	- Both malignant and infectious disease are FDG-avid	FDG-PET/CT: 100%/90.7% FDG-PET/MRI: 85.7%/100%
Ultrasound	- No exposure to ionizing radiation - Noninvasive - Fast - Patient friendly - Widely available	- Chance of not depicting the whole organ - Not suitable for whole-body imaging	- Diffuse disease is hard to detect - Inter-observer variation	N/A

^a^Most commonly in lymphoma imaging: skull base to mid-femur and including T1-weighted MRI and/or T2-weighted MRI and/or diffusion-weighted MRI

*CT* computed tomography, *FDG-PET* 18F-fluoro-2-deoxy-D-glucose positron emission tomography, *MRI* magnetic resonance imaging, *N/A* not applicable

**Table 2:** Criteria for extranodal Hodgkin lymphoma involvement

	Whole-body MRI	CT	FDG-PET	Ultrasound
Bone marrow	- Focal hypointensity on T1-weighted images and hyperintensity on T2-weighted images compared to muscle - Restricted diffusion on diffusion-weighted imaging	- N/A	- More than 2 PET positive lesions in skeleton (irrespective of positivity in CT or MRI) - FDG uptake bone marrow should be above FDG uptake in liver	- N/A
Bone		- Lytic/sclerotic appearance of the cortical bone		
Liver	- Hyperintense focus (less hyperintense than liquor) discrete from lymph node mass on T2-weighted image - Restricted diffusion on diffusion-weighted imaging	- Hypoattenuating nodules	- Focal PET positive lesions (confirmed by CT, MRI or US)	- One or more solid hypoechoic masses
Pleura	- Nodal lesion in pleura or chest wall - With or without pleural effusion^a^	- Pleural plaques or nodules	- PET positive pleural nodules	- N/A
Lung	- At least one intrapulmonary nodule >1 cm, not attached to lymph node mass - Or more than two nodules >2 mm and <10 mm within the lungs - Restricted diffusion on diffusion-weighted imaging	- Mass or mass-like consolidation - Parenchymal nodules - Peribronchial thickening - Alveolar/interstitial infiltrates	- PET positive nodules/masses	- N/A
Spleen	- Discrete nodules - With or without enlargement - No restricted diffusion on diffusion-weighted imaging	- Hypoattenuating nodules	- Focal PET positive lesions	- One or more focal hypoechoic abnormalities (irrespective of PET result)
E-lesion	- Disease infiltration per continuum from a lymph node mass into extralymphatic structures or organs			

^a^Pleural effusion alone is no indication for stage IV disease

*CT* computed tomography, *FDG-PET* 18F-fluoro-2-deoxy-D-glucose positron emission tomography, *MRI* magnetic resonance imaging, *N/A* not applicable

## Bone and bone marrow

**Fig. 1:**
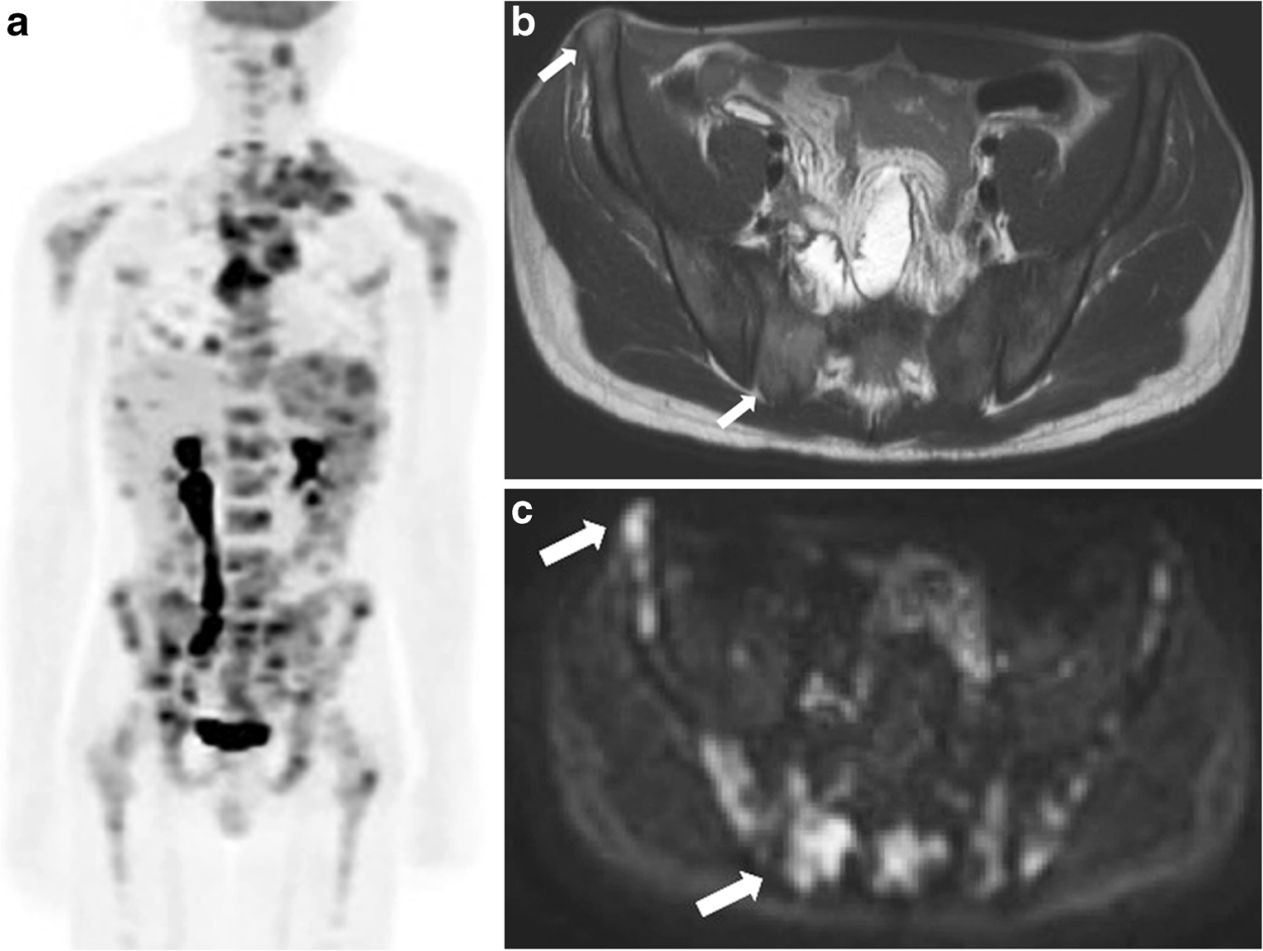
A 16-year-old boy with stage IV Hodgkin lymphoma with diffuse multifocal bone marrow involvement. **a** Coronal maximum intensity projection of the 18F-fluoro-2-deoxy-D-glucose positron emission tomography (FDG-PET) illustrates the extension of disease throughout the body, showing cervical and mediastinal affected lymph node stations as well as diffuse multifocal involvement in the skeleton. **b-c** Axial T2-weighted (TR/TE 895/80 ms) magnetic resonance image (**b**) shows areas of pathological increased T2 signal in ilium and sacrum (*arrows*) with corresponding restricted diffusion (**c**)

**Fig. 2:**
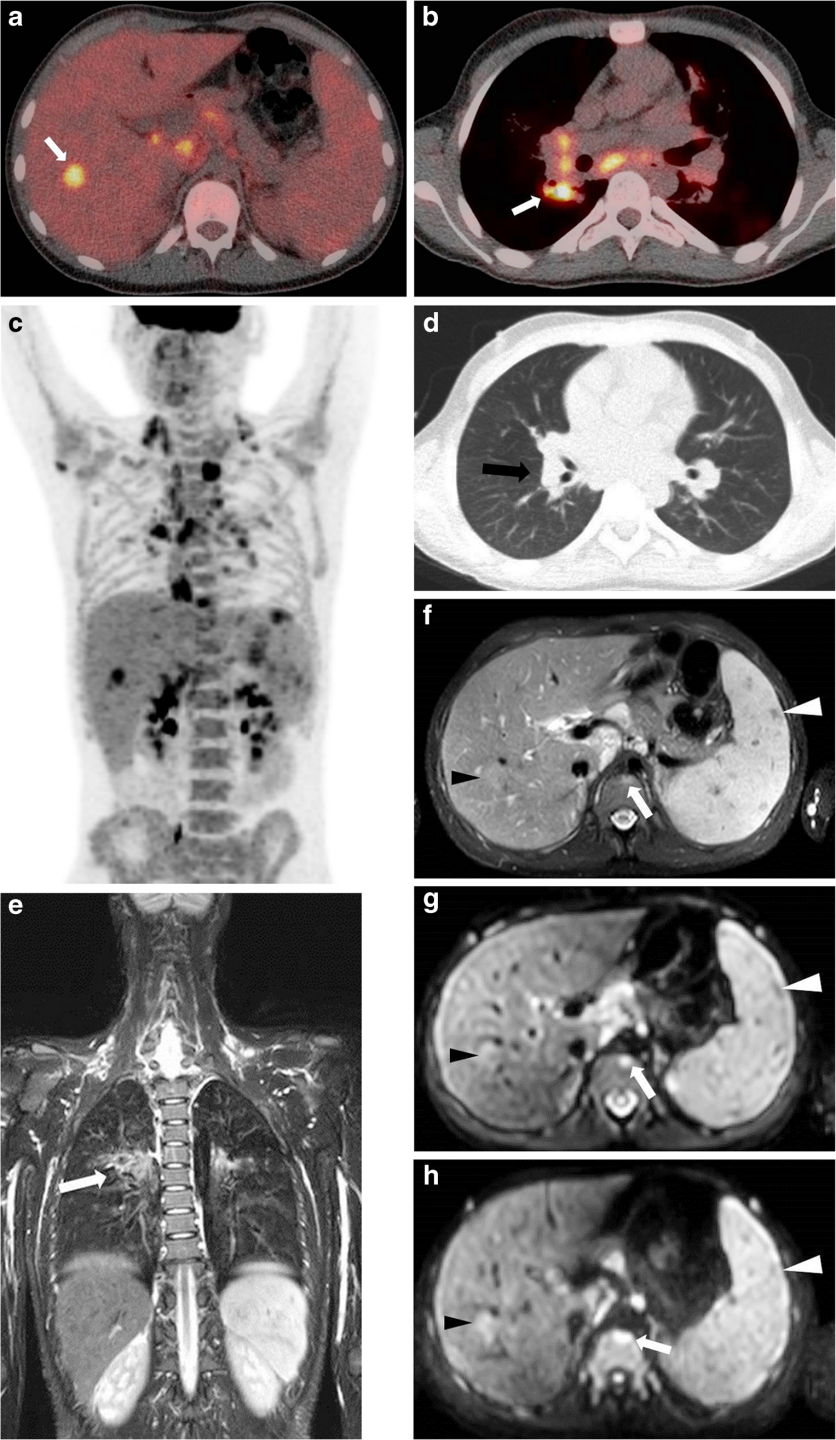
A 14-year-old boy with stage IV Hodgkin lymphoma with involvement of bone marrow, liver, lung (E-lesion) and spleen. **a-b** Axial 18F-fluoro-2-deoxy-D-glucose (FDG) positron emission tomography (PET)/computed tomography (CT) shows focal uptake of FDG in the liver (**a**) and the right lung (E-lesion originating from hilum/mediastinum) (**b**) (*arrows*). **c** Coronal maximum intensity projection of the FDG-PET illustrates the extension of disease throughout the body showing liver, splenic and pulmonary involvement as well as various lymph node regions both above and below the diaphragm. **d** Axial CT image shows an E-lesion in the right lung originating from the hilum/mediastinum. **e** Coronal T2-weighted magnetic resonance image (TR/TE 2,414/65) depicting the E-lesion in the right lung (*arrow*). **f** Axial T2-weighted image (TR/TE 2,414/65 ms) shows a focal hyperintensity in T12 vertebra (*arrow*), hypointense splenic lesions (*white arrowhead*) and a hyperintense hepatic lesion (*black arrowhead*). **g-h** Axial diffusion-weighted images at b=100 (**g**) and b=800 (**h**) illustrate that the focal liver lesion (*black arrowheads*) and the focal bone marrow lesion (*arrows*) show increasing hyperintensity with increasing b-values, indicating restricted diffusion. The focal splenic lesions appear hypointense on both b-values (*white arrowheads*)

**Fig. 3:**
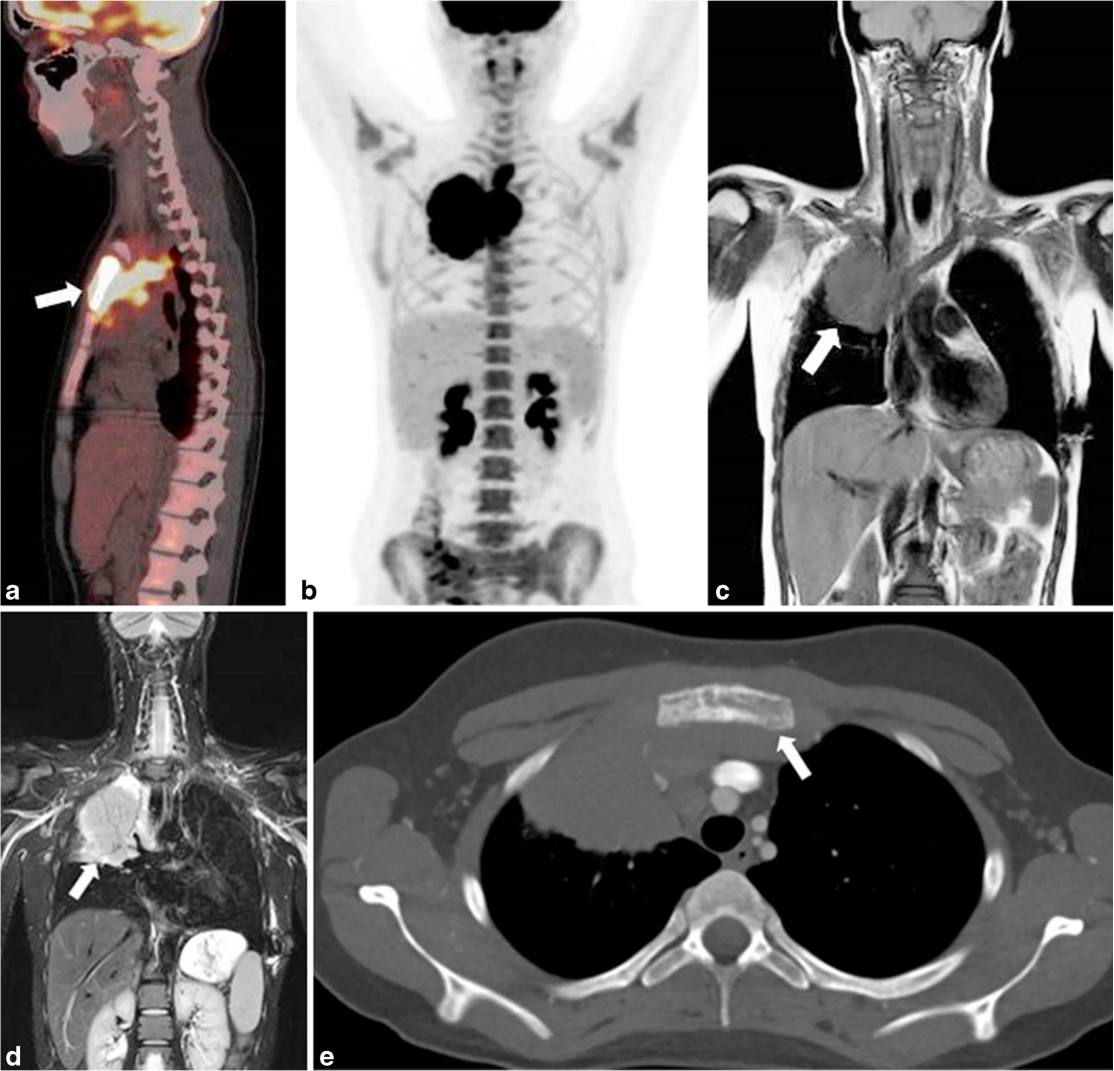
A 17-year-old girl with stage IIE Hodgkin lymphoma with an E-lesion in the sternum originating from the superior mediastinum. **a** Sagittal 18F-fluoro-2-deoxy-D-glucose (FDG) positron emission tomography (PET)/computed tomography (CT) shows elevated FDG uptake in mediastinum, and sternum (*arrow*). **b** Coronal maximum intensity projection of the FDG-PET illustrates an overview of the extension of disease throughout the body, showing no other extranodal involvement or affected lymph node stations. There is diffuse homogeneous activity of the bone marrow without focalities. Bone marrow biopsy was negative, therefore this diffuse activity is considered to be based on reactive bone marrow. **c-d** Coronal T1-weighted (TR/TE 537/17.5 ms) (**c**) and T2-weighted (TR/TE 2,414/65 ms) (**d**) magnetic resonance images show the mass in mediastinum and right lung (*arrows*). **e** Axial CT image depicts the E-lesion, with destruction of the sternal cortex (*arrow*)

Bone marrow is involved in 14% of children diagnosed with Hodgkin lymphoma [16] (Figs. 1 and 2). As for all extranodal disease sites, bone marrow involvement indicates stage IV disease. Until recently, bone marrow biopsy was part of standard care [2]. An advantage of bone marrow biopsy is the histopathological evidence of disease infiltration. However, important disadvantages are its invasive nature, sampling errors when the spread of disease is focal and, although rare, possible complications [17]. The current guidelines recommend FDG-PET/CT for assessing bone marrow involvement in paediatric Hodgkin lymphoma [2]. Bone marrow involvement is suspected when FDG-PET/CT positive lesions are seen, regardless of positivity on CT or MRI [2, 18]. A recent review showed that whole-body MRI might be a promising radiation-free alternative for detecting skeletal metastases of solid tumours in the paediatric population [19]. In addition, in an adult Hodgkin lymphoma study, whole-body MRI and FDG-PET/MRI were recently shown to have as comparable a diagnostic value as FDG-PET/CT for detecting bone marrow involvement [10, 17, 20, 21]. At FDG-PET/CT, bone marrow involvement appears as either diffuse or focal increased uptake in the bone marrow [13, 22, 23]. In untreated patients, bone marrow uptake above liver uptake is suggestive for bone marrow involvement [22]. Diffuse uptake of FDG in bone marrow not exceeding liver uptake is therefore considered paraneoplastic bone marrow activation rather than bone marrow infiltration [24] (Fig. 3). At whole-body MRI, bone marrow involvement shows relative low signal intensity on T1-weighted images compared to muscles, relatively high signal on T2-weighted images and restricted diffusion on diffusion-weighted imaging [17].

**Fig. 4:**
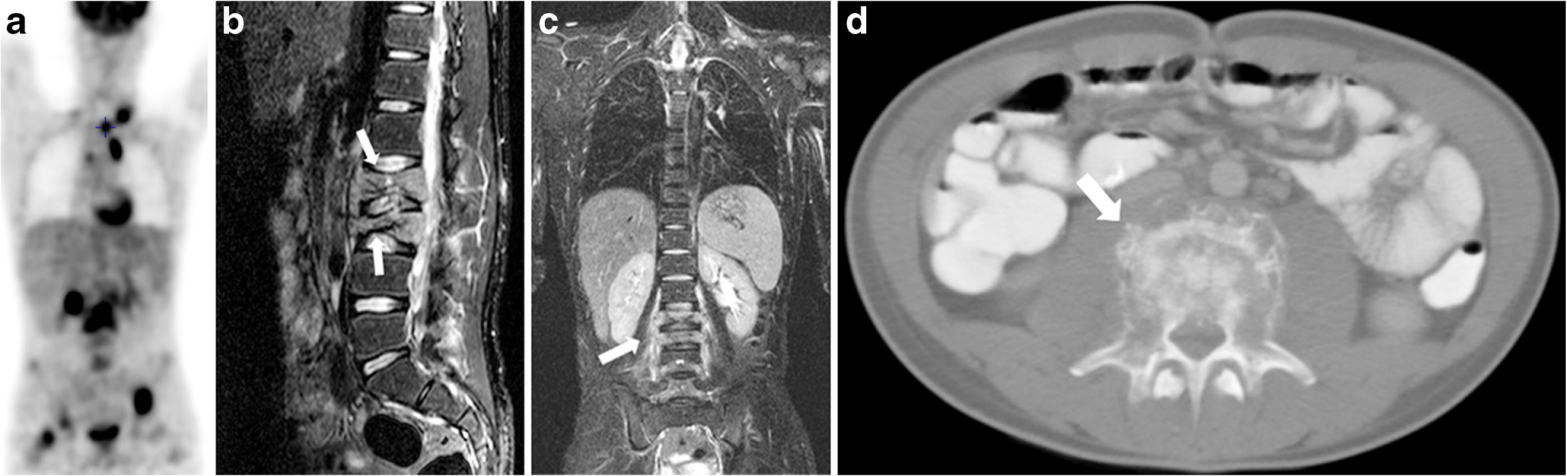
A 16-year-old boy with stage IV Hodgkin lymphoma with involvement of lumbar vertebrae 2 and 3 with extension (E-lesion) to surrounding muscle (psoas) and other tissues. **a** Coronal maximum intensity projection of the 18F-fluoro-2-deoxy-D-glucose positron emission tomography (FDG-PET) illustrates the extension of disease throughout the body, showing cervical and mediastinal affected lymph node stations as well as several locations in the skeleton (e.g., pelvis, head of humerus left, right acetabulum). **b** Sagittal T2-weighted (TR/TE 3,500/120 ms) magnetic resonance image shows pathological increased T2 signal with height loss of the vertebrae (*arrows*) and extension of the disease in the surrounding tissues. **c** Coronal T2-weighted (TR/TE 2,453/64 ms) image shows involvement of lumbar vertebrae 2 and 3 with lymphoma extension into the surrounding tissues (*arrow*). **d** Axial contrast-enhanced CT shows cortical destruction of the third lumbar vertebra (*arrow*)

The bone itself is affected in up to 4% of (adult) patients at presentation (Figs. 3 and 4); therefore, osseous involvement is much less common than bone marrow involvement [14]. Prognosis of patients with bone involvement is less favourable compared to those with bone marrow involvement [14]. The imaging features of bone involvement at conventional imaging, whole-body MRI and CT in Hodgkin lymphoma show bone destruction in an aggressive pattern [13]. FDG-PET combined with CT is the current modality of choice [2].

## Liver

The occurrence of liver involvement at presentation is 3%, according to literature [25], and in most of these cases the spleen is involved as well [12]. Hepatic involvement in lymphoma manifests usually as small or large focal lesions (Fig. 2). Hepatomegaly might be present, but liver size can be normal despite infiltration and a noninvolved liver might be enlarged. Small lesions are more common than large masses [13, 26]. Conglomerates of lymph nodes in the porta hepatis and retroperitoneum are commonly present as well [13]. On whole-body MRI, lesions usually appear hypointense on T1-weighted images and hyperintense compared to surrounding non-affected liver tissue on T2-weighted images (however, not as hyperintense as water or fluid, which would indicate a cyst rather than a tumour). Hepatic Hodgkin lymphoma shows diffusion restriction on diffusion-weighted imaging. FDG-PET/CT will show patchy foci of FDG uptake. It should be noted that liver FDG uptake is physiological, therefore identifying liver involvement on FDG-PET/CT can be challenging. On US and contrast-enhanced CT, lesions are hypoechoic and hypodense, respectively [22, 27, 28]. Given that the capability of whole-body MRI (including diffusion-weighted imaging) might be superior to FDG-PET/CT at detecting small lesions, combining FDG-PET and whole-body MRI was recently reported as promising for diagnosing liver lesions in adults [10, 29]. FDG-PET/MRI was shown to be at least equivalent to FDG-PET/CT in staging lymphoma with the additional benefit of radiation dose reduction [10, 29]. Depending on the child’s age and size and the use of either a diagnostic CT and/or a dose-reduced CT for the FDG-PET/CT examination, estimated relative dose reductions for PET/MRI are reportedly between 48% and 73%, according to the recent literature [5, 30]. The current European guidelines for paediatric Hodgkin lymphoma state that focal FDG-PET positive lesions should be confirmed by contrast-enhanced CT, MRI or US to diagnose hepatic lymphoma involvement [2]. In addition, abdominal US is still considered part of standard care to assess both focal liver and splenic involvement.

## Lung and pleura

**Fig. 5:**
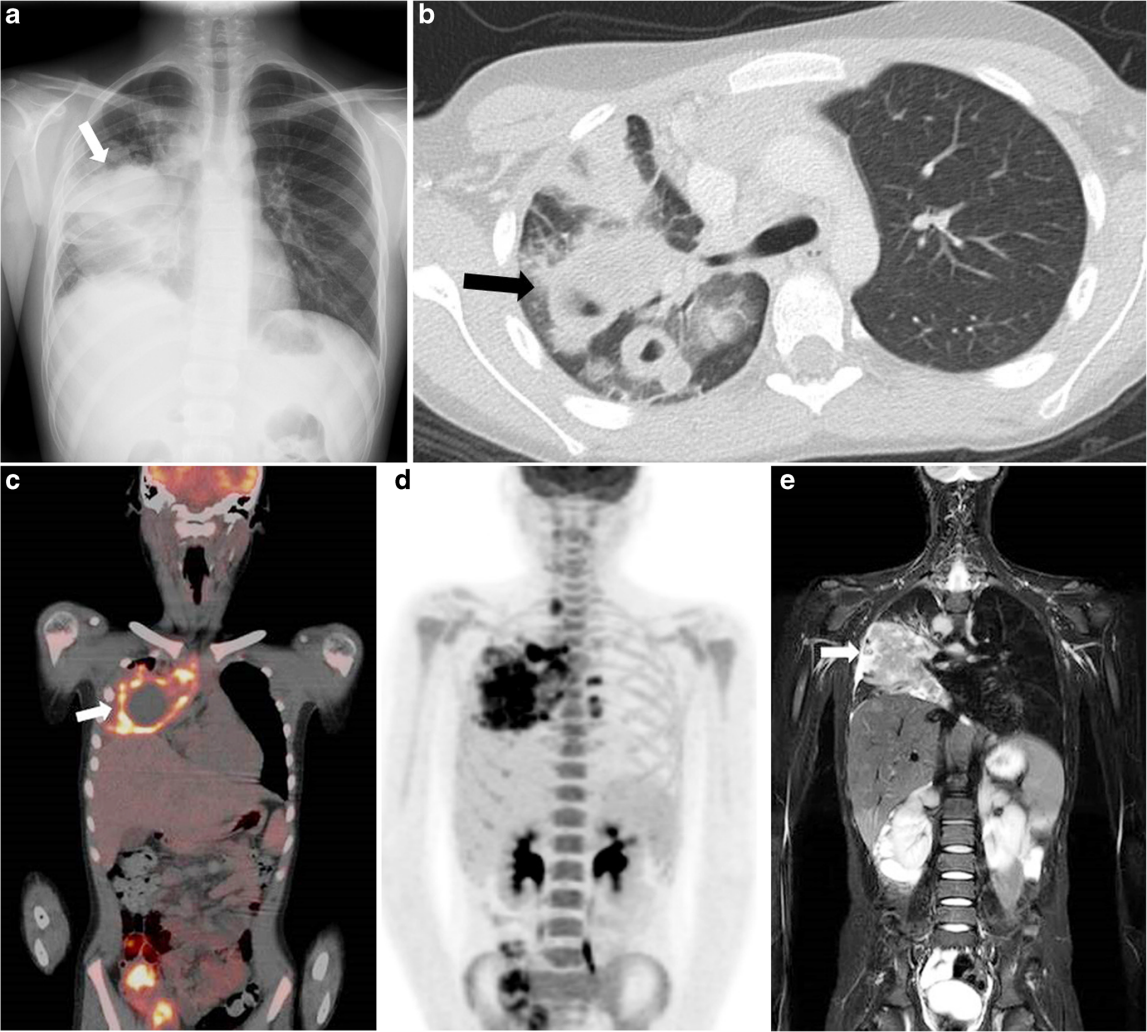
A 13-year-old boy with stage IV Hodgkin lymphoma with pulmonary disease involvement. **a** Conventional radiography of the thorax depicts at least one large pulmonary mass (*arrow*). **b** Axial computed tomography (CT) shows the intrathoracic masses with cavitations (*arrow*). After conventional radiography and CT, the child was first treated with antibiotics. When symptoms persisted, a biopsy revealed the diagnosis of Hodgkin lymphoma. **c** Coronal 18F-fluoro-2-deoxy-D-glucose (FDG) positron emission tomography (PET)/computed tomography (CT) shows elevated FDG uptake in the right hemithorax with multiple cavitations (*arrow*). **d** Coronal maximum intensity projection of the FDG-PET illustrates the extension of disease throughout the body. Apart from the pulmonary lesion and hilar and cervical lymph nodes, no other extranodal sites are affected. Physiological FDG uptake in genitourinary tract and bowels is seen. **e** Coronal T2-weighted (TR/TE 2,414/65 ms) magnetic resonance image shows an intrathoracic mass (*arrow*) and hepatomegaly

Pulmonary parenchymal involvement occurs in up to 12% of children with Hodgkin lymphoma, with the common findings being pulmonary masses, nodules and cavitations [31, 32] (Figs. 2 and 5). Pulmonary involvement is reported to be more common in the paediatric than adult population, and can present as primary disease, E-lesion or stage IV disease [32]. Strikingly, as primary pulmonary Hodgkin lymphoma can manifest in multiple ways, there is commonly a delay in diagnosis due to not instantly considering cancer in children. The two more common forms in which pulmonary Hodgkin lymphoma presents are as a pulmonary E-lesion (an extranodal extension of disease originating from mediastinal and/or hilar affected lymph nodes) and pulmonary stage IV disease. In the case of stage IV disease, the extranodal extension is due to haematological metastasis, not through connected nodal sites or via lymphogenous spread. Distinguishing stage IV pulmonary disease from a pulmonary E-lesion can be diagnostically challenging. Especially when a pulmonary nodule close to a pulmonary E-lesion is found it can be hard to determine whether the nodule is a separate stage IV lesion or belongs to the nearby E-lesion.

The current modality of choice is CT [2, 23]. A recent study compared MRI of the lung with CT for detecting pulmonary nodules, stating that MRI might provide an alternative imaging modality for pulmonary nodules [33]. It is known that MRI of the lung is challenging due to respiratory and cardiac motion artefacts and the low signal-to-noise ratio [33]. Over the past years, MRI sequences have been improved and dedicated lung MRI is able to detect nodules with high diagnostic accuracy [33]. However, at whole-body MRI, which is a recent modality of interest for paediatric Hodgkin lymphoma, this accuracy is most likely lower due to less dedicated sequences. Unfortunately, the use of whole-body MRI as well as FDG-PET/MRI to detect pulmonary lesions specific for Hodgkin lymphoma has not yet been investigated, as FDG-PET/MRI, in particular, might be of additive value in the diagnostic process by combining the high image quality and low radiation exposure of whole-body MRI with the high sensitivity of FDG-PET.

Pleural effusion is relatively common in Hodgkin lymphoma [34]. The amount of effusion is usually small and a solitary pleural effusion is not classified as pleural involvement. Distinction between this nonmalignant pleural effusion, contagious disease and rare pleural involvement of Hodgkin lymphoma is difficult but an important component of staging [2, 34]. Hodgkin lymphoma of the pleura manifests as plaques or nodules and might, given the limited sensitivity of CT to identify pleural involvement of malignancies [35], be undervalued on CT alone. Therefore, the addition of FDG-PET is useful [26]. Pleural lymphoma involvement manifests as hypointense plaques and/or nodules on both T1-weighted and T2-weighted MRI and shows restricted diffusion on diffusion-weighted images [36].

## Spleen

**Fig. 6:**
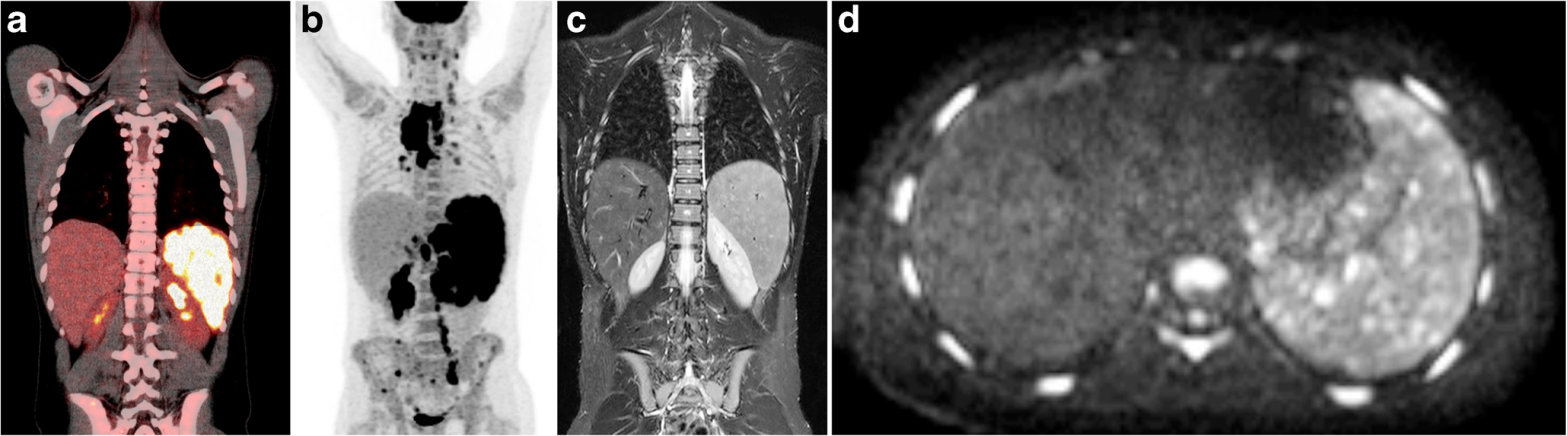
A 13-year-old boy with stage III Hodgkin lymphoma with diffuse splenic disease involvement. **a** Coronal 18F-fluoro-2-deoxy-D-glucose (FDG) positron emission tomography (PET)/computed tomography (CT) shows diffuse splenic FDG uptake and splenomegaly. **b** Coronal maximum intensity projection of the FDG-PET shows the extension of disease throughout the body including diffuse splenic infiltration and involvement of various lymph node stations (cervical, mediastinal, para-aortal). **c** Coronal T2-weighted magnetic resonance image (TR/TE 5,231/65 ms) shows diffuse splenic inhomogeneity and splenomegaly. **d** Axial diffusion-weighted image (b=800) shows diffuse inhomogeneity of the spleen

Although splenic involvement in Hodgkin lymphoma is classified as nodal disease [2], assessing the spleen is of great importance for staging Hodgkin lymphoma since it is the most common site of subdiaphragmatic nodal disease [37]. Involvement of the spleen indicates stage I disease, if solely affected. Splenic involvement accompanied by involvement of lymph node stations is classified as either stage II disease when only nodal stations below the diaphragm are affected, or stage III disease if the spleen and nodal stations above, or above and below, the diaphragm are affected [2]. In up to 16% of newly diagnosed adults, the spleen is involved [16]. Splenic involvement can be present in different patterns: enlargement of the spleen without focal lesions, a large solitary mass, multiple focal nodular lesions (Fig. 2) and diffuse infiltration with lesions smaller than 5 mm [38] (Fig. 6). Organ size alone should not be used to identify splenic lymphoma, since spleen size can be normal despite infiltration and a spleen without neoplastic involvement might be enlarged [26]. For the current gold standard imaging modality FDG-PET/CT, sensitivity and specificity for splenic involvement are 97% and 100%, respectively [38]. At FDG-PET/CT, splenic uptake above hepatic and bone marrow uptake is considered to be an indicator for splenic involvement [13, 39]. Recent literature showed that whole-body MRI is reasonably accurate concerning splenic involvement in lymphoma [40]. On MRI, focal involvement of the spleen appears as low T2 signal lesions with low signal intensity on diffusion-weighted imaging compared to its healthy surroundings. As most other benign and malignant splenic lesions appear as high T2 signal lesions [41], T2-weighted MR images are therefore helpful in distinguishing lymphoma from other nodules. However, diffuse infiltration might appear no different from normal splenic tissue on MRI [40, 42]. Nodules are typically hypoechoic at US, and at contrast-enhanced CT, lesions appear hypodense compared to surrounding normal splenic tissue [14]. Again, diffuse infiltration is usually not detected with US or CT [14]. The European guidelines for imaging in paediatric Hodgkin lymphoma state that splenic involvement should be assumed if FDG-PET/CT positive lesions are confirmed by CT, MRI or US or if multiple small focal changes in the spleen structure are detected and suspicious for tumour, irrespective of the FDG-PET/CT result [2].

## Other extranodal manifestations

**Fig. 7:**
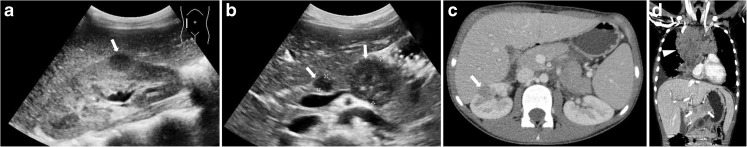
A 16-year-old boy with stage IV Hodgkin lymphoma with involvement of the pancreas and the right kidney. **a** Ultrasound image of the right kidney shows a hypoechoic renal lesion (*arrow*). **b** Ultrasound image of the pancreas depicts two hypoechoic pancreatic lesions (*arrows*). **c** Axial computed tomography (CT) image depicts a hypodense Hodgkin lymphoma lesion in the right kidney (*arrow*). **d** Coronal CT image shows two hypodense lesions in the pancreas (*arrows*) indicating pancreatic lymphoma infiltration. A large mediastinal mass is seen as well (*arrowhead*)

As stated earlier, Hodgkin lymphoma may affect every tissue or organ. The incidence of extranodal involvement of most organs other than those discussed so far (bone marrow, liver and lungs) is reportedly low [14]. In adults, Hodgkin lymphoma rarely involves the central nervous system, genitourinary tract (including kidneys), muscles, gastrointestinal tract, heart and pericardium, which is in accordance with our clinical experience in children [14]. Involvement of the central nervous system may affect all central nervous system areas (e.g., brain, meninges, spinal cord and cauda equina), but intraspinal lesions occur more frequently than intracranial lesions [14]. Central nervous system involvement is most often a late manifestation of extended disease. To detect central nervous system involvement, MRI has a greater sensitivity and specificity for both meninges, spinal cord and cauda equina compared to CT [14]. Lesions in the spinal cord appear hyperintense on T2-weighted MR images. Diagnostic accuracy of FDG-PET is low for detection of central nervous system involvement due to high physiological FDG uptake [13]. Genitourinary tract involvement (Fig. 7) is seldomly seen in Hodgkin lymphoma and most commonly involves the kidneys presenting as either perirenal infiltration or focal lesions [43]. Renal lesions can be difficult to depict at FDG-PET due to renal physiological excretion of FDG. MRI could therefore be of additional diagnostic value. Renal involvement on MRI appears as nodules that are hypointense on T1-weighted images, hyperintense on T2-weighted images and with signs of restricted diffusion on diffusion-weighted imaging. Muscle involvement is in most cases seen as paravertebral masses originating from retroperitoneal lymph nodes or originating from bone involvement (Fig. 4) and should be regarded as an E-lesion [14]. Hodgkin lymphoma of the gastrointestinal tract is rare compared to non-Hodgkin lymphoma and usually involves a single site. The stomach is the most frequent site presenting on imaging as gastric wall thickening [14]. Cardiac or pericardial involvement of Hodgkin lymphoma presents as thickening of the epicardium and/or pericardium or as nodular masses in the pericardium [14, 44]. Although rare, one should be aware of the fact that spread of disease might occur in these infrequently affected organs at staging and during follow-up of paediatric Hodgkin lymphoma.

## Conclusion

This pictorial essay describes the spectrum of imaging features of extranodal involvement in paediatric Hodgkin lymphoma at different imaging modalities. Extranodal disease occurs in a minority of children, but if present it has great impact on prognosis and treatment strategy. The most common sites of extranodal involvement in paediatric Hodgkin lymphoma include bone marrow, liver and lungs. FDG-PET/CT and whole-body MRI are the imaging methods of choice in children with Hodgkin lymphoma, whereas US is being used as a radiation-free, patient-friendly option to evaluate intra-abdominal organ involvement.
